# Observation of hapten-induced sensitization responses for the development of a mouse skin sensitization test, including the elicitation phase

**DOI:** 10.1038/s41598-022-24547-1

**Published:** 2022-11-18

**Authors:** Ryo Kamata, Yukiko Okawa, Yuto Hamaguchi, Soma Tabata, Masanori Terasaki, Kazuki Takeda

**Affiliations:** 1 Laboratory of Toxicology, School of Veterinary Medicine, Kitasato University, 35-1 Higashi 23-Bancho, Towada-Shi, Aomori, 034-8628 Japan; 2 Environmental Chemistry Laboratory, Graduate School of Arts and Sciences, Iwate University, 3-18-34 Ueda, Morioka, Iwate 020-8550 Japan; 3 Department of Computer Science, Tokyo Institute of Technology, 4259-J3-1818, Nagatsuta-cho, Midori-ku, Yokohama, Kanagawa 226-0026 Japan; 4 Laboratory of Toxicology, Department of Environmental Veterinary Sciences, Graduate Faculty of Veterinary Medicine, Hokkaido University, N18 W9, Kita-ku, Sapporo, 060-0818 Japan

**Keywords:** Skin manifestations, Allergy, Reverse transcription polymerase chain reaction

## Abstract

The only official method that can detect the skin sensitizing potential of chemicals, including the elicitation response, is the OECD test guideline (TG) 406. However, this guideline uses guinea pigs, which requires complex procedures. Since a simple and complete test method for evaluating skin sensitization is needed, especially for mechanistic studies of skin sensitization, this study confirmed the reactivity of mice to skin sensitizing substances. We set up a protocol involving one induction exposure of the test substance to the back skin, followed by three challenge exposures to the auricle (Protocol 2), and compared their skin sensitization responses with the results of two exposures to the auricle and back skin every 2 weeks (Protocol 1) and a local lymph node assay (TG442B). A hapten 2,4-dinitrofluorobenzene caused significant auricular thickening, skin inflammation, and enlarged auricular lymph nodes in Protocols 1 and 2. These changes were more pronounced in Protocol 2. Plasma IgE and IgG1 and gene expression of IL4, IFNγ, and perforin were significantly increased in Protocol 2. Cell proliferation in the auricular lymph nodes was observed in both protocols as in TG442B. These results indicate that Protocol 2 can be a good candidate for a relatively simple skin sensitization test.

## Introduction

Allergic diseases have been identified as one of the major health problems affecting a large number of people in developed countries and urban areas^1^. Although these issues are primarily caused by exposure to chemicals, not only in daily life or at work but also in the environment, their causative and exacerbation factors are often unknown. As a result, sensitizing skin diseases such as allergic contact and atopic dermatitis can significantly affect daily life, as they not only result in severe itching but also in bad appearance over a long period. Therefore, skin toxicity testing is deemed essential for the production of pesticides and other chemicals. The immune system is known to have a complex process in terms of skin exposure to chemicals causing skin sensitization (sensitizing substances or sensitizers), resulting in skin symptoms, such as erythema (redness), edema, and blisters. This complex establishment process of skin sensitization involves two major steps: induction by initial contact with a sensitizer and elicitation by subsequent contacts.

**Figure 1:**
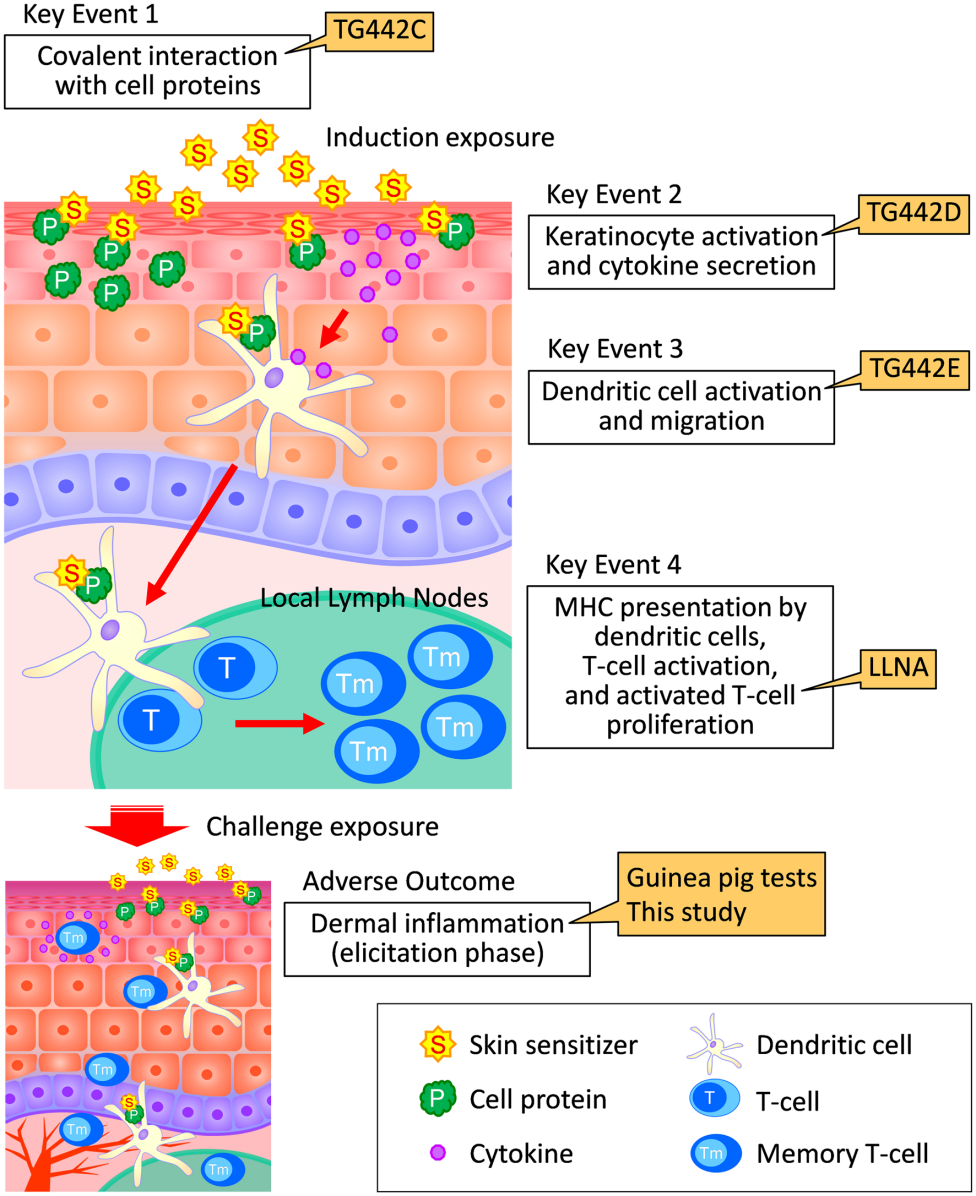
The OECD adverse outcome pathway (AOP) and test guidelines (TGs) for skin sensitization. *LLNA* local lymph node assay, *MHC* major histocompatibility complex.

The Organisation for Economic Co-operation and Development (OECD) has proposed several test methods (test guidelines, TGs) for detecting chemical substances that might cause skin sensitization. Among them are in vivo tests, such as TG406: Guinea Pig Maximization Test (GPMT) and Buehler Test using guinea pig^2^; TG429: Local lymph node assay (LLNA) using mice^3^; TG442A: LLNA modified by Daicel, based on ATP content (LLNA: DA)^4^; and TG442B: LLNA: 5-bromo-2′-deoxyuridine (BrdU)-enzyme-linked immunosorbent assay (ELISA)^5^. Specifically, while TG406 can detect responses from the induction phase of skin sensitization to elicitation phase, LLNA and its modified mouse tests can only detect induction. Additionally, current knowledge proposes a mechanism of skin sensitization, summarized as the adverse outcome pathway (AOP), from the early stages at the molecular level to the onset of adverse effects, namely, allergic contact dermatitis^6^ (Fig. 1). The OECD defines four key events in the AOP, namely, the covalent binding of electrophilic substances to nucleophilic centers in skin proteins, keratinocyte activation, dendritic cell activation, and T-cell proliferation, and has adopted several in vitro tests to evaluate these key events (Fig. 1). For example, TG442C^7^ is a test method that evaluates the first key event, the protein-sensitizer binding; TG442D^8^ evaluates the second key event, that is, keratinocyte activation; and TG442E^9^ evaluates the third key event, that is, dendritic cell activation. Additionally, LLNA is an in vivo test method that indirectly evaluates T-cell proliferation, which is part of the fourth key event. Unfortunately, the in vitro tests only detect each event in the AOP and cannot completely replace animal experiments. Also, although LLNA has the advantages of simplicity, shorter test duration, and less burden on animals compared to other in vivo testing methods, it can only evaluate the induction phase of skin sensitization. Therefore, although these test methods are useful for screening the skin sensitization of chemicals required to develop pharmaceuticals and cosmetics, they are deemed unsuitable for studying skin reaction mechanisms by sensitizers and detecting exacerbating factors in dermatitis because they do not consider skin reactions in the elicitation phase. Only TG406 was determined as the most viable method. Still, it uses guinea pigs, and it is a test method that can completely evaluate skin sensitization reactions. Besides, guinea pigs are relatively large as laboratory animals, and the test procedure is complicated. Hence, a test method using mice, which is the most popular laboratory animal, is required to easily evaluate skin sensitization phases from induction to elicitation.

**Figure 2:**
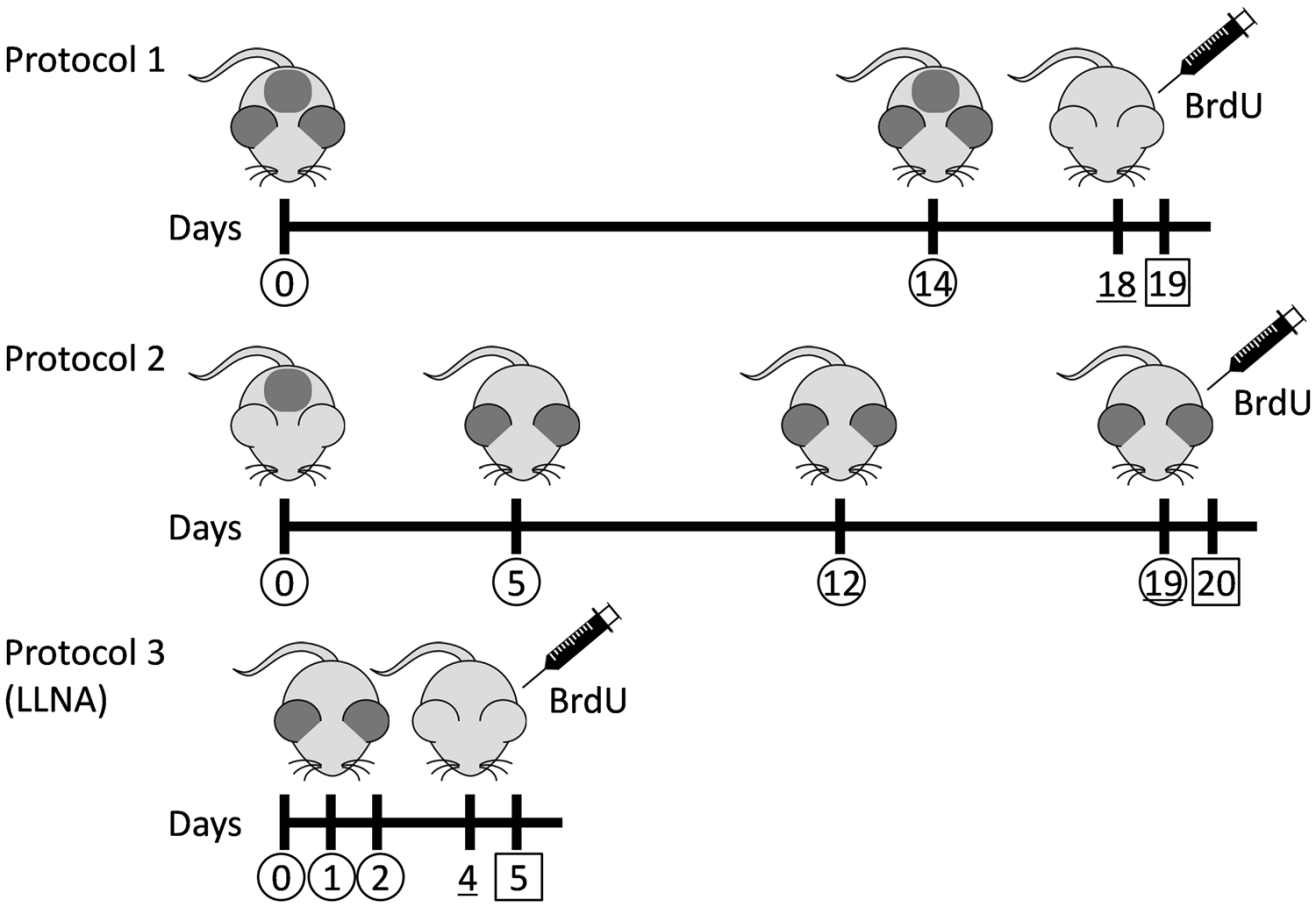
Exposure schedules and procedures for each protocol. Numbers circled, underlined, and squared represent exposure dates to the test substances, BrdU (5-bromo-2′-deoxyuridine) administration periods, and sample collection dates, respectively. The dark-colored areas of the mouse skin are the sites of exposure to the test substance. *LLNA* local lymph node assay (OECD TG442B). The numbers of animals used in Protocols 1, 2 and 3 were 8, 7 and 10 per group, respectively.

Based on the facts presented above, this study aimed to confirm the responsiveness of mice to the sensitizers. Our goal was to establish a simple and complete test method for detecting skin sensitization, which can be used for the toxicity evaluation of chemicals, including mechanistic studies on contact dermatitis. First, we set up two protocols (Fig. 2): two exposures of the test substance to the auricle and back skin every 2 weeks (Protocol 1) and one induction exposure to the back skin, followed by three challenge exposures to the auricle (Protocol 2). Then, skin sensitization responses due to exposure were evaluated on the basis of morphological changes at the application sites, plasma antibody levels, and gene expression in the skin of the auricle. Finally, cell proliferation of the auricular lymph nodes was measured and compared with those from OEDC TG442B.

## Results

In each protocol, no significant difference in the body mass of mice among all groups on any measurement day was observed (Supplementary Table S1 online). Furthermore, no noticeable symptoms or changes in appearance other than erythema of the auricle were observed in any protocol.

### Protocol 1: two sensitizing exposures, 2 weeks apart

**Figure 3:**
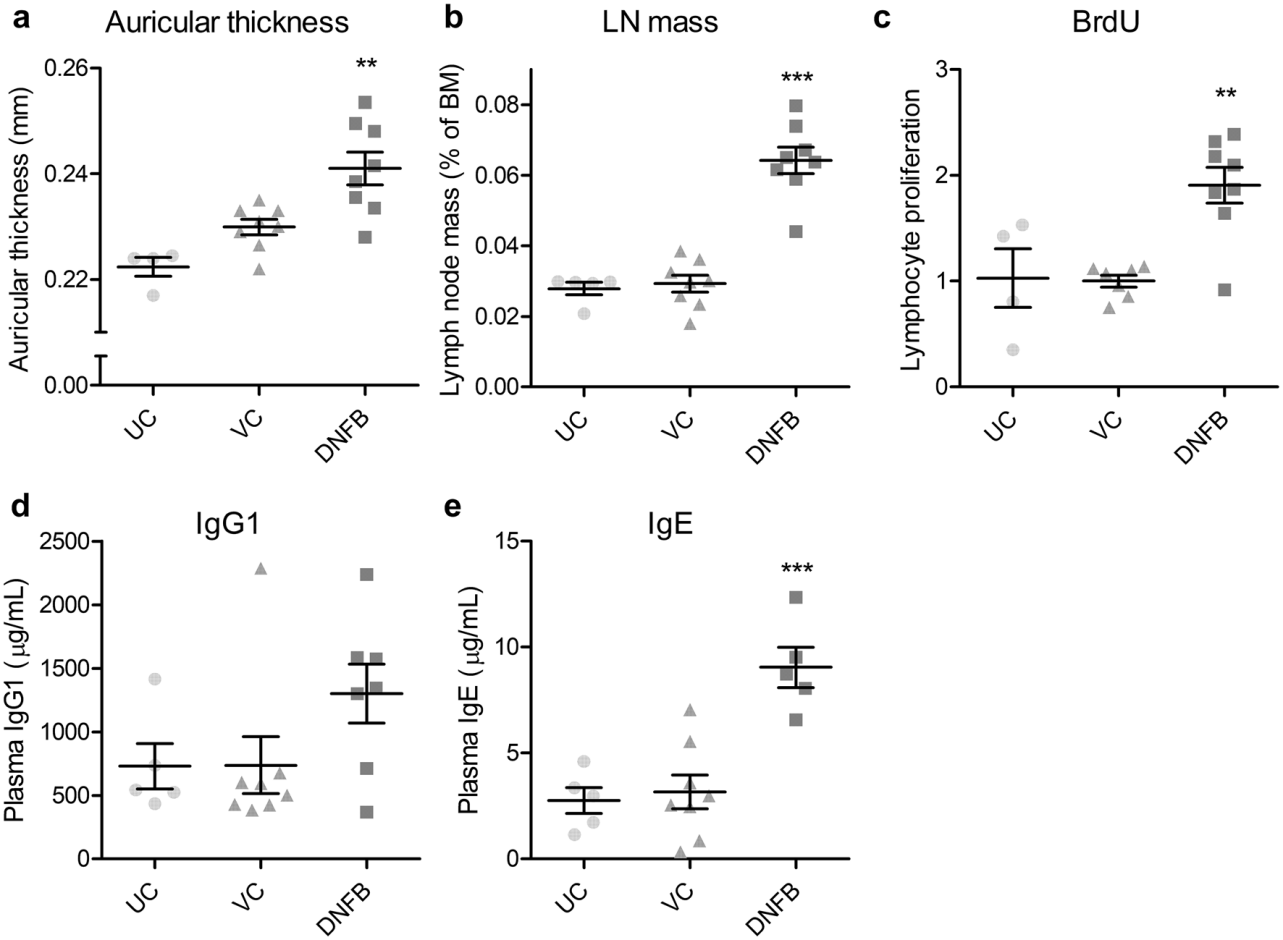
Changes in the auricle, auricular lymph nodes, and plasma immunoglobulins of mice after two doses of DNFB, 2 weeks apart. Left auricular thickness (**a**), auricular lymph node-to-body mass ratio (**b**), and BrdU-labeled lymphocyte proliferation in the lymph nodes (**c**, ratio to vehicle control), including plasma IgG1 (**d**) and IgE (**e**), were measured in the untreated (UC) and vehicle-only (VC) or DNFB-treated mice, 24 h after the second treatment. The graphs are scatter plots, where each symbol represents an actual measurement, and the centerlines and error bars represent the mean and standard error, respectively. Asterisks indicate levels of significant differences from the vehicle control: **P < 0.01, ***P < 0.001.

Twenty-four hours after the second application, the auricular thickness of 2,4-dinitrofluorobenzene (DNFB)-treated mice was noted to increase significantly by 4.8%, compared with the vehicle controls (Fig. 3a). Investigations also revealed that DNFB treatment significantly increased the auricular lymph node-to-body mass ratio and lymphocyte proliferation, as measured by BrdU-labeling in the lymph nodes by 119% and 90.6%, respectively, compared with the vehicle control (Fig. 3b,c). Moreover, DNFB significantly increased plasma IgE levels by 185% compared with the vehicle control (Fig. 3e), including IgG levels (76%, Fig. 3d). Furthermore, we observed that although the auricles of all DNFB-treated mice showed varied thickening of the epidermis, dermis, and subcutaneous tissue, accompanied by edema and hyperemia in the thickened tissue, these reactions were not observed in the back skin. Besides, while mast cells were observed in all groups by toluidine blue staining, no difference was noted in the number of mast cells between the exposed groups. Results also showed that while DNFB-treated mice exhibited ear- and back-scratching behavior 24 h after the second application, the frequency was insignificantly different from that of vehicle controls. No significant change was observed between the untreated and vehicle controls in any other measurements.

### Protocol 2: one induction and three challenges, 1 week apart

**Figure 4:**
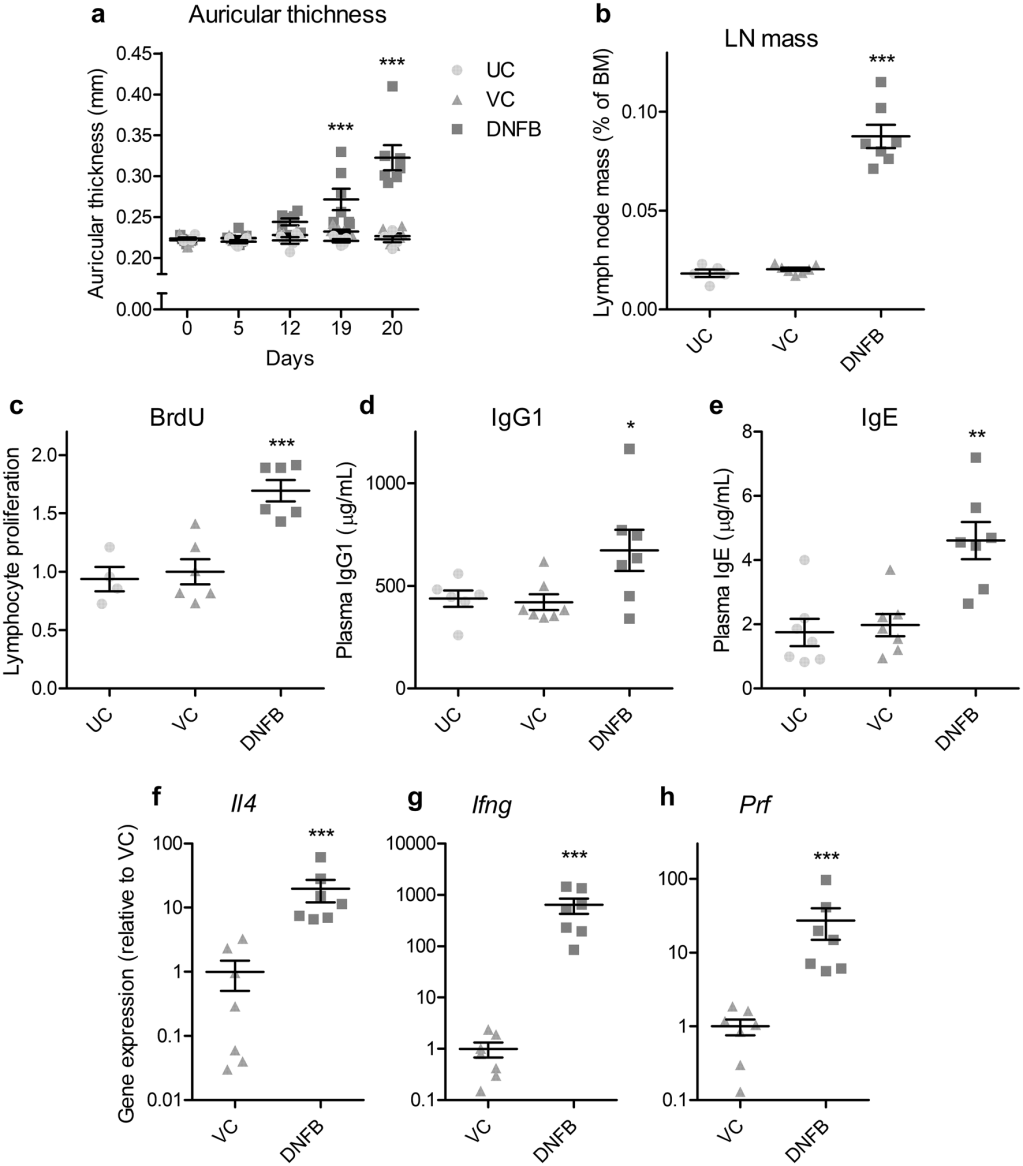
Changes in the auricle, auricular lymph nodes, plasma immunoglobulins, and auricular gene expression of mice after one DNFB induction and three challenges, 1 week apart. Left auricular thickness (**a**) was measured in the untreated (UC) and vehicle-only (VC) or DNFB-treated mice on days 0, 5, 12, 19, and 20 after induction. Auricular lymph node-to-body mass ratio (**b**) and BrdU-labeled lymphocyte proliferation in the lymph nodes (**c**, ratio to vehicle control), including plasma IgG1 (**d**) and IgE (**e**), were also measured 24 h after the final treatment. Transcript levels of *Il4* (**f**), *Ifng* (**g**), and *Prf* (**h**) in the auricle tissue of the vehicle-only (VC) or DNFB-treated mice were measured by real-time PCR using *Actb* as an internal control and expressed as ratios to the vehicle control. The graphs are scatter plots, where each symbol represents an actual measurement, and the centerlines and error bars represent the mean and standard error, respectively. Asterisks indicate levels of significant differences from the vehicle control: *P < 0.05, **P < 0.01, ***P < 0.001.

**Figure 5:**
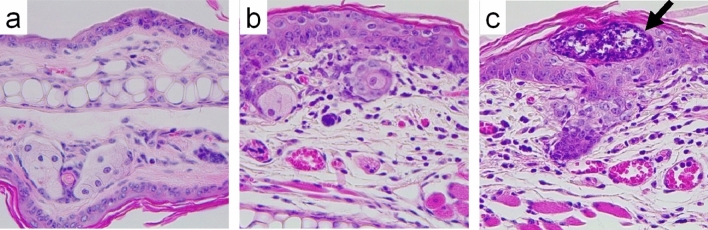
Representative histological changes in the auricular skin from mice after one DNFB induction and three challenges, 1 week apart. No pathological changes were observed in vehicle control mice (**a**). The epidermis, dermis, and subcutaneous tissue of the specimens were thickened, and edema, hyperemia, and infiltration of inflammatory cells were observed in the thickened tissues (**b**). The arrow shows microabscesses in the epidermis (**c**). The histological changes observed in (**b**) and (**c**) were confirmed in all DFNB-treated mice (n = 7).

Investigations revealed that the auricular thickness of DNFB-treated mice increased as the number of treatments also increased. Specifically, we observed significant increases of 16.9% on day 19 of the experiment (1 week after the second application and just before the third application) and 42.3% on day 20 (before blood collection) compared with that of vehicle controls (Fig. 4a). Results also showed that DNFB treatments significantly increased the auricular lymph node-to-body mass ratio and BrdU-labeled lymphocytes in lymph nodes by 330% and 69.5%, respectively, compared with the vehicle control (Fig. 4b,c). Moreover, it significantly increased plasma IgG and IgE levels by 59.8% and 133%, respectively, compared with the vehicle control (Fig. 4d,e). However, no significant change was observed between the untreated and vehicle controls in these measurements. Additionally, although obvious erythema was observed in the auricles of all DNFB-treated mice but not in those of the untreated control or the vehicle control, the frequency of the ear-scratching behavior 24 h after the last application was not significantly different between the DNFB-treated and vehicle control groups. Furthermore, while DNFB significantly increased the expression of cytokine and related genes in auricular tissue samples, *Il4*, *Ifng*, and *Prf* in DNFB-treated mice were 19.7, 637, and 27.3 times higher than those in the vehicle control group (Fig. 4f–h). Investigations also revealed that in all DNFB-treated mice, HE-stained auricle specimens showed signs of an allergic inflammatory response. Notably, the epidermis, dermis, and subcutaneous tissue were thickened, and edema, hyperemia, and infiltration of inflammatory cells were observed in the thickened tissue samples (Fig. 5b). In addition, microabscesses were observed in the epidermis (Fig. 5c). However, these pathological changes were not observed in the untreated or vehicle-treated mice (Fig. 5a). After toluidine blue staining, although mast cells were observed in all groups, no difference was noted in the number of mast cells between the exposed groups.

**Figure 6:**
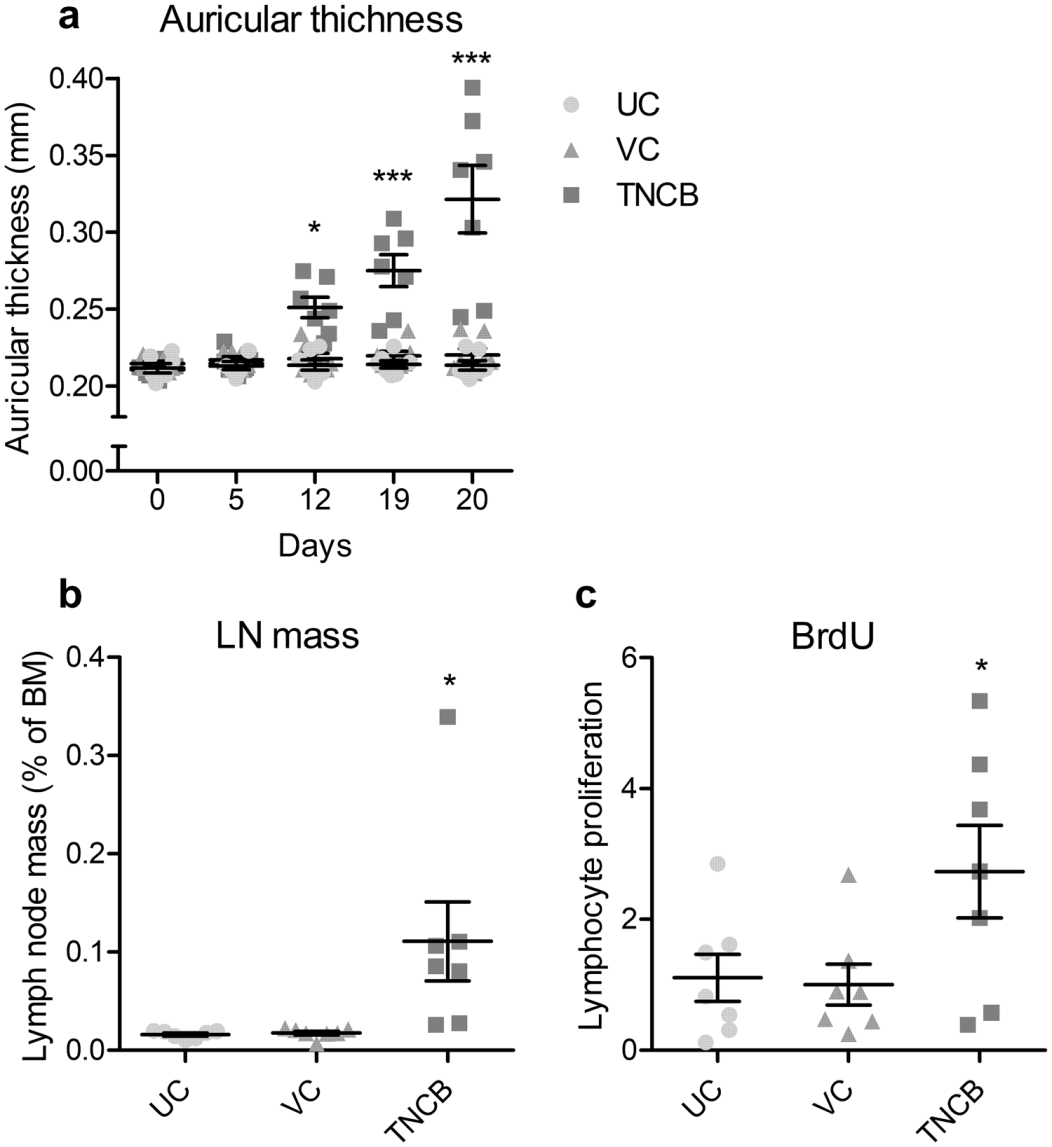
Changes in the auricle and auricular lymph nodes of mice after one TNCB induction and three challenges, 1 week apart. Left auricular thickness (**a**) was measured in the untreated (UC) and vehicle-only (VC), or TNCB-treated mice on days 0, 5, 12, 19, and 20 after induction. Auricular lymph node-to-body mass ratio (**b**) and BrdU-labeled lymphocyte proliferation in the lymph nodes (**c**, ratio to vehicle control) were measured 24 h after the final treatment. The graphs are scatter plots, where each symbol represents an actual measurement, and the centerlines and error bars represent the mean and standard error, respectively. Asterisks indicate levels of significant differences from the vehicle control: *P < 0.05, ***P < 0.001.

Alternatively, results showed that while the 2,4,6-trinitrochrolobenzene (TNCB) treatment significantly increased the auricular thickness of mice, increasing by 25.1% on day 19 and 45.7% on day 20 of the experiment compared with the vehicle control (Fig. 6a), it also significantly increased the auricular lymph node-to-body mass ratio and BrdU-labeled lymphocytes in lymph nodes by 532% and 173%, respectively, compared with the vehicle control (Fig. 6b,c). In the auricular tissue of all TNCB-treated mice, the epidermis, dermis, and subcutaneous tissue were thickened, with characteristic edema, hyperemia, and inflammatory cell infiltration being observed in the thickened tissue. Nevertheless, there were no microabscesses as observed in DNFB.

### Protocol 3: local lymph node assay (OECD TG 442B)

**Figure 7:**
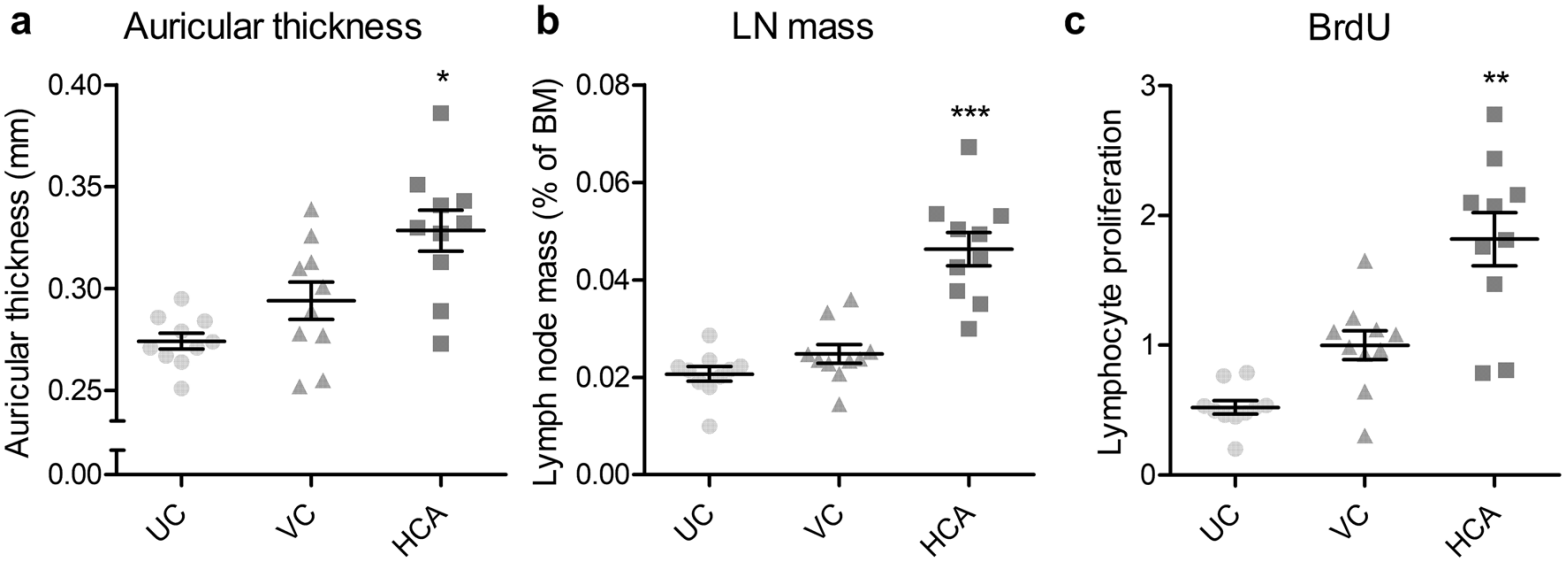
Changes in the auricle and auricular lymph nodes of mice treated with HCA, once daily for 3 days, according to OECD test guideline 442B. Left auricular thickness (**a**), auricular lymph node-to-body mass ratio (**b**), and BrdU-labeled lymphocyte proliferation in the lymph nodes (**c**, ratio to vehicle control) were measured in the untreated (UC) and vehicle-only (VC) or DNFB-treated mice, 3 days after the final treatment. The graphs are scatter plots, where each symbol represents an actual measurement, and the centerlines and error bars represent the mean and standard error, respectively. Asterisks indicate levels of significant differences from the vehicle control: *P < 0.05, **P < 0.01, ***P < 0.001.

According to the OECD TG 442B, we measured auricular thickness and lymphocyte proliferation by BrdU-labeling in the auricular lymph node. Three days after the last application, the auricular thickness of α-hexyl cinnamaldehyde (HCA)-treated mice increased significantly by 11.7% compared with the vehicle controls (Fig. 7a). Similarly, HCA treatment has significantly increased the auricular lymph node-to-body mass ratio and lymphocyte proliferation by 86.8% and 81.8%, respectively, compared with the vehicle control (Fig. 7b,c). However, no obvious erythema in the auricles of any three groups (HCA-treated group, the untreated group, or the vehicle control group) and no significant changes in any other measurements were observed.

## Discussion

Although skin sensitization tests are currently being replaced by alternative methods that do not use laboratory animals for animal welfare, in vitro and in silico tests only represent a part of skin sensitization and cannot reproduce the entire process, even in multiple combinations. Moreover, the GPMT and Buhler test described in TG406 (the only official methods that can detect skin sensitization, including the elicitation phase) require complicated procedures such as intradermal injections of test substances and multiple applications of closed patches^2^. Additionally, because the positive determination of the test is based solely on gross observation of skin reactions, quantitative evaluation is impossible. Therefore, the Mouse Ear Swelling Test (MEST) has been reported as a test method for detecting elicitation reactions. Many chemicals were also evaluated for this assay in the 1980s and 1990s^10,11^. In MEST, chemicals are applied multiple times (generally three times) to the abdomen and auricular skin as sensitizing exposures, followed by a challenge exposure to the auricle on day 10. Then, the elicitation response is evaluated by measuring auricular swelling. However, while MEST brings detection sensitivity challenges, the OECD protocol states that chemicals with negative results in this test or LLNA should be adopted in the guinea pig tests^12^. As a result, LLNA and its variants are currently used as alternatives to the guinea pig tests. LLNA has the advantages of simplicity and shorter study duration compared to in vivo testing methods such as guinea pig tests and MEST. In particular, LLNA can quantitatively detect skin sensitization reactions as cell proliferation in lymph nodes. The test period is very short: 5 days for LLNA^4^, compared to approximately 2 weeks, 4 weeks, and 1 month for MEST, GPMT, and Buhler^11,12^, respectively. Furthermore, LLNA does not induce skin symptoms, so it is less burdensome to the animals. Still, LLNA only detected the induction phase of skin sensitization, not the skin response in the elicitation phase. Therefore, these tests cannot be used for advanced judgments like final determination of skin sensitization, mechanistic studies of skin reactions caused by sensitizers, and the detection of exacerbating dermatitis factors. Since allergic inflammatory responses were observed in the auricular skin tissue in Protocol 2 of this study, including edema, hyperemia, inflammatory cell infiltration, and microabscesses, it can be concluded that this assay was able to reproduce the elicitation phase. Quantitative measurement of each test item as in this study is expected to facilitate the evaluation of the skin sensitization potential of chemicals. It was feared that the inflammatory changes in the auricular skin would cause a greater burden on the mice than LLNA. However, since no behavioral changes due to itching or pain were observed, it appears that the burden caused by the allergic reaction was kept at a fairly low level.

In this study, both induction and challenge exposure to haptens caused mice to develop skin symptoms characteristic of the elicitation phase of contact dermatitis. We set up Protocol 1, applying the test substance twice to wider areas of the skin (auricle and back). Then, Protocol 2 was set up, applying the test substance thrice, only to the auricle, as challenge exposures after one induction exposure. Notably, the hapten doses used in this study were set at low concentrations within the concentration range where positive responses could be expected based on the OECD test guidelines or previously reported results. In a previous study using guinea pigs, DNFB was used as a positive control, usually at 0.1–0.5% concentrations, and 0.1% DNFB produced mild redness (score 1–2) after elicitation exposure^13^. Although the haptens used here were different, the auricular skin thickening and lymph node hypertrophy in Protocol 1 were similar to those of LLNA. Furthermore, no gross skin symptoms were observed in either experiment. Meanwhile, in Protocol 2, although lymphocyte proliferation was similar to that in Protocol 1 and LLNA, auricular skin thickening and lymph node hypertrophy were more pronounced. While no officially recommended test method exists using mice to determine the elicitation phase of skin sensitization, a number of studies have been conducted on the development of test methods using mice and the mechanism of the elicitation phase. Alternatively, auricular thickening of approximately just under 30% was observed 48 h after exposure to 0.15% DNFB in MEST^14^. Previously, Yamashita et al.^15,16^ developed a test method with an elicitation phase of one challenge exposure, 7 days after induction with test substances for 3 consecutive days, usually performed in LLNA (this dosing schedule is similar to the MEST). Then, they detected the elicitation effects of several chemicals. In this method, DNFB was observed to be positive for elicitation at a dose of 0.25%. Additionally, at 0.13% of 2,4-dinitrochlorobenzene (DNCB), which has similar skin sensitivity to DNFB, although the mass of the lymph nodes increased, erythema of the auricular skin was slight, and the thickness of the auricle remained unchanged. Only the highest dose at 0.50% showed a marked change in auricular thickness. Similarly, since significant auricular thickening occurred after a second challenge exposure at 0.15% DNFB and was enhanced after the third in this study, it is important to consider the frequency and interval of exposures to properly trigger the elicitation phase. In another study, Kitamura et al.^17^ set several exposure periods and frequencies to create a model of atopic dermatitis and evaluated allergic reactions, particularly from increased serum IgE levels. They applied 100 μL of 0.15% DNFB to the back skin once a week, three times, or once every 2 weeks, twice in total. Their investigations showed skin symptoms such as redness and edema on days 15–19 and a significant increase in IgE on day 19. The significant changes appeared after day 15, regardless of the number of exposures, consistent with our results of auricular thickening. Thus, we propose a sensitization period of about 2 weeks to form the elicitation phase.

In studies of allergic contact dermatitis, the murine contact hypersensitivity (CHS) model, which can reproduce both induction and elicitation phases, is frequently used. However, studies using CHS have focused on exploring the pathogenesis of contact dermatitis and examining the efficacy of dermatitis medications^18–20^, and have rarely been used to assess chemical toxicity. Therefore, its usefulness must be verified before it can be used for toxicity assessment. CHS usually consists of a single induction exposure to the sensitizer on the abdominal skin, followed by challenge exposure on the auricular skin once 5 days later (acute model), daily for 3 days (subacute) or daily for about 10 days (chronic)^20,21^. In contrast, in MEST, induction exposure is daily for 3 or 4 days, followed by one challenge exposure 5 days later^14^. In CHS, only one challenge exposure thickened the auricle, but did not increase lymph node weight or IgE antibodies and caused few histological changes^20,21^. Therefore, it appears that three challenges, as in this study, are necessary to form clear elicitation response. While the reported CHS studies used high concentrations of haptens that reliably cause contact dermatitis for those purposes^20^, our method showed skin reactions at lower concentrations, those proposed in the OECD. Although it has the disadvantage of a long exposure period, sufficient skin reactions have occurred, and the regular weekly treatments will reduce the burden on both the mice and the administering staff.

In Protocol 2, the gene expression of *Il4*, *Ifng*, and *Prf* in the auricular tissue was noted to be significantly increased by DNFB application. Yamanishi et al.^22^, to study the mechanism of atopic dermatitis, applied 100 μL of 3% oxazolone (4-ethoxymethylene-2-phenyl-2-oxazolin-5-one) to the abdomen of C57BL/6J mice as an induction exposure, followed by 10 μL of 0.5% oxazolone to the auricle every other day as challenge exposures. Results showed that while *Ifng* expression in the auricular skin significantly increased from 2 days after the first challenge exposure, *Il4* significantly increased from 4 days later. In particular, increases in *Il4* expression (more than 1000 times that before the challenge) were remarkable compared to *Ifng* (10–30 times), contrary to our *Il4* and *Ifng* expression results. Because IL-4 and IFN-γ are cytokines produced primarily by Th2 and Th1 cells, respectively, and perforin is a cytolytic protein produced by cytotoxic T cells and natural killer (NK) cells, it is considered that DNFB activates humoral immunity by Th2 cells and activates Th1 cells to enhance cell-mediated immunity by cytotoxic T and NK cells. Specifically, it has also been observed that while perforin was increased in the skin of patients with contact dermatitis and those with atopic dermatitis, it was expressed in both CD4+ and CD8+ T cells^23,24^. Based on these facts, and since the results of our Protocol 2 showed marked *Ifng* and significant *Prf* increases, we consider that the exposure schedule for this protocol should reproduce at least Th1-cell-mediated contact dermatitis.

Blood IgG1 and IgE levels increased in Protocols 1 and 2 (although IgG1 in Protocol 1 was insignificant). Similarly, Kitamura et al.^17^ showed an increase in IgE levels from 5 days after the second exposure. Furthermore, in the oxazolone exposure, serum IgG1 and IgE levels also increased from the 8th day after the challenge exposure^22^. Therefore, although no mast cell migration was observed in skin tissue in this study, considering the increased *Il4* expression in the auricular skin, it is still suggested that Th2 cell differentiation occurred by challenge exposure to haptens.

Species differences are one of the concerns that arise when using laboratory animal models, and it is necessary to verify that humans and model animals respond similarly. Similar to the present study, it has been observed in humans that chemical allergens induce both Th2 and Th1-type responses; Dhingra et al.^25^ performed gene array and RT-PCR analyses on patch-tested human skin samples and reported that gene expression, especially of Th2-related cytokines, increased by fragrance. Although chemical-specific IgG and IgE have been used to identify the causative agents of allergy, the elevation of total IgG1 and IgE in blood from exposure to a single chemical has not been reported in humans, so the hapten-induced antibody elevation observed in this or other mouse studies is unlikely to replicate the human antibody response^26^. Nevertheless, the high antibody production capacity of mice may be useful for sensitive detection of chemical sensitization. Since elevations in blood total IgE have been observed in humans for complete antigens such as house dust mite and pollen^27,28^, the reactivity of mice to haptens may be stronger than that of humans.

In this study, although we detected several changes attributed to skin sensitization reactions in Protocol 2, we did not test the dose dependence of the test. Since the challenge exposure to hapten has developed responses that were considered to induce cell-mediated immunity by Th1 cells and humoral immunity by Th2 cells, this system is expected to help reproduce contact dermatitis based on the participation of these two immune response systems. We set the challenge exposure to once a week, three times, to easily and reliably cause skin sensitization reactions with the elicitation phase. Still, twice may be sufficient, considering the results of auricular thickening. Although the test period is longer than that of CHS and MEST, the number of treatments remains the same. The exposure method and intervals are also clear and constant, making it a useful test method. Therefore, to establish a reliable skin sensitization test, it is necessary to consider detection factors and confirm dose dependence.

## Methods

### Animals

Specific pathogen-free female BALB/cByJJcl or CBA/J mice at 7 weeks of age were purchased from CLEA, Japan (Tokyo, Japan) or the Charles River Laboratories, Japan (Yokohama, Japan), respectively. BALB/c mice were used in the experiments because their large reticuloendothelial organs facilitate lymph node handling (information from the breeder) and are therefore frequently used in allergy research^18^, and CBA/J mice are the strain recommended in the OECD regional lymph node studies^3–5^. In addition, in sensitization studies using mice, young adult female mice are usually used to eliminate the effects of aggression, which is a concern in males; females are also used in the LLNA and MEST^11^. They were housed under conventional conditions: 20–26 °C room temperature and 40–60% humidity, with a 12 h light and dark cycle. Standard rodent chow (CE-2, CLEA Japan) and water were freely available. All animal experimentations were conducted according to the protocol approved by the president of Kitasato University after reviews by the Institutional Animal Care and Use Committee (permission number: 18-081, 19-002, and 19-187) in accordance with relevant guidelines and regulations. Reporting of experiments involving animals follows the recommendations of the ARRIVE guidelines.

### Chemicals

We purchased DNFB and TNCB from the Tokyo Chemical Industry (Tokyo, Japan), whereas HCA was purchased from Wako Pure Chemical Industries (Osaka, Japan). Then, the chemicals were dissolved in a recommended vehicle solution, acetone/olive oil (4:1, v/v)^5^, to prepare test solutions of 0.15% DNFB, 0.1% TNCB, and 25% HCA.

### Experimental design

#### Protocol 1: two sensitizing exposures, 2 weeks apart

After a 1-week acclimation period, the back skin of mice was shaved under isoflurane anesthesia; then, the back skin and the dorsal skins of both ears were tape-stripped to remove the stratum corneum. One hundred μL and 20 μL of 0.15% DNFB or the vehicle solution were applied to the back skin and the ear’s skins, respectively (Fig. 2). Two weeks after the first application, mice were subjected to a similar treatment. Then, 4 days after the second sensitization (on day 18), 0.5 mL of 10 mg/mL BrdU solution was intraperitoneally injected to detect lymphocyte proliferation induced by DNFB, followed by a blood sample collection through cardiac puncture under anesthesia 24 h later. Finally, the animals were monitored to detect hypersensitivity reactions.

#### Protocol 2: one induction and three challenges, 1 week apart

After 1-week acclimation, 100 μL of 0.15% DNFB or the vehicle was applied to the shaved back skin of mice. On days 5, 12, and 19 after induction, 20 μL DNFB or the vehicle was applied to the dorsal skin of both ears. Then, the BrdU solution was intraperitoneally injected on day 19. Twenty-four hours later, blood samples were collected, after which animals were monitored to detect hypersensitivity reactions. The same administration experiment was conducted for 0.1% TNCB.

#### Protocol 3: local lymph node assay (OECD TG442B)

An OECD test guideline for determining skin sensitization (TG442B)^5^ was conducted to compare the lymph nodes and immune system responses in Protocols 1 and 2, thereby detecting allergic contact sensitization. One week after acclimation, 25 μL of 25% HCA or the vehicle was applied once daily to the dorsal skin of both ears for 3 days. Then, 2 days after the third application, the BrdU solution was intraperitoneally injected. Blood samples were finally collected 24 h later, after which animals were surveyed to detect hypersensitivity reactions.

### Irritation of the ear auricle

Before anesthesia was administered for blood collection in Protocols 1 and 2, the scratching behavior of mice was recorded under an observation chamber for 15 and 30 min, respectively, using a digital video camera (Nikon D5600, Nikon Corporation, Tokyo, Japan). We observed that mice scratched their auricles multiple times, using their hind paws in one movement. Therefore, one attempt with multiple scratching behavior was counted as one event.

Additionally, before the first treatment in Protocol 1 and each treatment in Protocol 2, the thickness of the auricle was measured using a micrometer gauge (Soft-Touch Micro CLM, Mitutoyo, Kawasaki, Japan) and before blood collection in Protocols 1, 2, and 3.

### Lymphocyte proliferation

The draining auricular lymph nodes of mice were bilaterally excised to detect cell proliferation by measuring BrdU uptake during DNA synthesis. According to the OECD TG442B^5^, cell suspensions were prepared from the lymph nodes. After the lymph nodes had been mashed in phosphate-buffered saline (PBS, pH 7.4) containing 10 mM ethylenediaminetetraacetic acid, 2 mM benzylsulfonyl fluoride, 0.1 mg/mL soybean trypsin inhibitor, 1.0 mg/mL bovine serum albumin, and 40 g/mL gentamicin, the solution was passed through a plastic pestle, pluriStrainer 200 μm (pluriSelect Life Science, Leipzig, Germany), after which the volume of filtered cells was adjusted to 15 mL with PBS. Next, cell proliferation in the resulting single-cell suspensions was determined using a Cell Proliferation ELISA, BrdU (colorimetric) (Roche Diagnostics GmbH, Mannheim, Germany) with peroxidase-conjugated anti-BrdU antibody, according to the manufacturer’s protocol. The absorbance of BrdU-labeled samples was measured using a spectrophotometer at 450 nm, using a reference wavelength of 620 nm. Cell proliferation induced by the sensitizer was calculated as the absorbance ratio in the treated groups to that in the concurrent vehicle control group.

### Immunoglobulins

Plasma was obtained by centrifuging the collected blood and stored at − 80 °C until analysis. The plasma’s total IgE and IgG1 levels were measured using an IgE Mouse Uncoated ELISA Kit and an IgG1 Mouse Uncoated ELISA Kit (Thermo Fisher Scientific, MA, USA), respectively, according to the manufacturer’s protocols.

### Histopathology

Pieces of the back skins from mice in Protocol 1 and right auricles in Protocol 2 were removed, fixed in 10% phosphate-buffered formalin, and then embedded in Pathoprep568 (Fujifilm Wako Pure Chemical Corporation, Tokyo, Japan), following routine methods. The paraffin blocks were cut into thin sections (4 μm) and stained with hematoxylin and eosin (HE) for pathological examinations or toluidine blue to detect mast cells. Pathological examinations of the tissue preparations were performed using a light microscope.

### Cytokine and related gene expression

Left auricles in Protocol 2 were stored in an RNAlater™ Stabilization Solution (Thermo Fisher Scientific) at − 30 °C until analysis. Total RNA was extracted from the left auricles using a Monarch Total RNA Miniprep Kit (New England BioLabs, MA, USA), according to the manufacturer’s instructions. Subsequently, the RNA was reverse-transcribed to cDNA using a LunaScript RT SuperMix Kit (New England BioLabs). Next, following the manufacturer’s guidelines, quantitative real-time PCR was performed using a Luna Universal qPCR Master Mix (New England BioLabs). Two microliter of cDNA (40 ng) was amplified with 500 nM each of the specific primer combinations for the target genes and 10 μL Luna Universal qPCR Master Mix (New England BioLabs) in a total volume of 20 μL. PCR conditions were as follows: initial denaturation of 95 °C for 60 s, 40 cycles of 95 °C for 15 s, and 60 °C for 30 s. Then, real-time PCR was conducted for each sample according to a relative standard curve method, using a StepOnePlus™ Real-Time PCR System (Thermo Fisher Scientific). Standard curves were obtained from serially diluted sample mixtures, after which the expression levels of samples were measured using these standard curves. Primers for the respective target genes (listed in Supplementary Table S2 online) were designed to anneal at 60 °C.

### Statistics

Statistical analyses were conducted using GraphPad Prism 5 for Windows, version 5.04 (GraphPad Software, Inc., CA, USA). Differences between the control and treatment groups were tested using the Dunnett’s multiple comparison test or Student’s *t* test. Differences between the control and treatment groups in auricular thickness measurements in Protocol 2 were tested using a two-way repeated-measures ANOVA with Bonferroni multiple comparisons. Results were expressed as means ± SEM, and statistical significance was noted when *P* < 0.05.

## Supplementary Information

Supplementary Table S1.

Supplementary Table S2.

## Data Availability

All relevant data are within the paper and its Supplementary Information files.
